# Sex- and Exercise-Dependent Modulation of Hypertrophic Remodeling by the MCT1 rs1049434 Polymorphism

**DOI:** 10.3390/genes17020188

**Published:** 2026-02-02

**Authors:** Natalia Fernández-Suárez, María Teresa Viadero, Teresa Amigo, José Antonio Benitez-Muñoz, Rocío Cupeiro, Domingo González-Lamuño

**Affiliations:** 1 Pediatric Cardiology Division, University Hospital Marqués de Valdecilla, 39008 Santander, Spain; natalia.fernandezs@scsalud.es (N.F.-S.); mteresa.viadero@scsalud.es (M.T.V.); 2 Laboratory of Pediatrics, School of Medicine, Universidad de Cantabria, 39011 Santander, Spain; teresa.amigo@unican.es; 3 Facultad de Salud y Deporte, Universidad Alfonso X el Sabio–Mare Nostrum, 29001 Málaga, Spain; joseantonio.benitez.munoz@upm.es; 4 LFE Research Group, Department of Health and Human Performance, Faculty of Physical Activity and Sport Science (INEF), Universidad Politécnica de Madrid, 28040 Madrid, Spain; 5 Pediatric Division, University Hospital Marqués de Valdecilla, University of Cantabria, 39008 Santander, Spain

**Keywords:** hypertrophic cardiomyopathy, sarcomeric variants, MCT1 (*SLC16A1*), rs1049434, lactate transport, metabolic remodeling, exercise, sex differences

## Abstract

Background: The monocarboxylate transporter 1 (MCT1) plays a central role in myocardial lactate handling and metabolic adaptation. The functional rs1049434 polymorphism (T1470A; Asp490Glu) affects MCT1-mediated lactate transport and substrate utilization, but its clinical relevance in sarcomere-related hypertrophic cardiomyopathy (HCM) remains poorly defined. Methods: We studied 56 carriers of pathogenic or likely pathogenic sarcomeric variants examined in a familial HCM program. All participants underwent standardized clinical phenotyping, including electrocardiography, transthoracic echocardiography, and cardiac magnetic resonance imaging. Genotyping of MCT1 rs1049434 was performed on genomic DNA. Analyses focused on sex-stratified genotype distribution, phenotypic expression among the 26 individuals who fulfilled diagnostic criteria for HCM, and the influence of habitual vigorous exercise. Septal wall thickness was the primary structural endpoint. Results: Among the 26 patients with established HCM (10 women, 16 men), a marked sex-specific effect emerged. Female carriers of the T-allele (TT/TA) exhibited significantly greater interventricular septal thickness compared with AA homozygotes (23.2 vs. 14.2 mm; *p* = 0.037). In men, septal thickness did not differ by genotype. However, male patients engaged in vigorous physical activity showed a consistently milder structural phenotype, including lower septal thickness (18.3 vs. 19.9 mm; *p* = 0.585) and directionally favorable markers of mechanical severity. Phenotypic distribution was predominantly asymmetric septal hypertrophy in both sexes, without genotype-dependent differences. Conclusions: The phenotypic impact of MCT1 rs1049434 in sarcomere-positive HCM is context-dependent. In women, impaired monocarboxylate handling is associated with greater hypertrophic remodeling, whereas in men, exercise-related metabolic conditioning appears to attenuate disease severity. These findings support a genotype–sex–environment interaction relevant to precision medicine approaches in HCM.

## 1. Introduction

Hypertrophic cardiomyopathy (HCM) is a genetically determined myocardial disorder characterized by unexplained left ventricular hypertrophy—most commonly involving the interventricular septum—and associated with diastolic dysfunction, arrhythmias and heart failure [1,2]. Beyond sarcomeric mechanical dysfunction, increasing evidence supports a central role for altered myocardial energy metabolism in disease progression. The human HCM myocardium demonstrates enhanced glycolytic flux, reduced fatty acid oxidation and perturbations in redox balance, changes that contribute directly to maladaptive hypertrophic remodeling [3,4,5].

Monocarboxylate transporter 1 (MCT1), encoded by the *SLC16A1* gene, facilitates the transmembrane transport of lactate and other monocarboxylates, enabling metabolic coupling between glycolytic and oxidative tissues through the lactate shuttle [6,7]. Among the multiple single-nucleotide polymorphisms described within *SLC16A1*, the common rs1049434 variant (T1470A; Asp490Glu) has been the most extensively characterized from a functional standpoint. This polymorphism affects MCT1-mediated lactate transport and has been consistently associated with altered lactate kinetics, substrate utilization, and increased glycolytic strain during exhaustive exercise [8,9,10,11].

Although several additional MCT1 variants have been linked to interindividual differences in lactate production and clearance in athletic populations, most show limited functional annotation or context-dependent effects, often emerging only in large, performance-based cohorts or under specific experimental conditions (e.g., high-intensity anaerobic protocols) [12]. In contrast, rs1049434 has repeatedly demonstrated robust associations across independent cohorts and experimental settings, supporting its prioritization as a biologically relevant modifier of lactate handling. While these effects have been characterized primarily in skeletal muscle, their relevance to myocardial remodeling and energetic stress in genetically determined cardiomyopathies remains uncertain.

Sex-related differences in cardiac metabolism and mitochondrial reserve are increasingly recognized and may modulate the phenotypic expression of inherited heart disease [5]. In parallel, habitual vigorous exercise induces metabolic conditioning, enhancing mitochondrial oxidative capacity and lactate utilization efficiency [13,14,15]. We therefore hypothesized that the metabolic consequences of the MCT1 rs1049434 variant may influence the expression of sarcomere-positive HCM in a sex-dependent manner, and that regular vigorous physical activity could further modify this relationship.

Accordingly, the aims of this study were to evaluate: (i) the sex-stratified distribution of MCT1 rs1049434 genotypes in a cohort of carriers of pathogenic sarcomeric variants; and (ii) the impact of this polymorphism on structural disease expression among patients with established HCM, considering biological sex and habitual vigorous exercise as key modifying factors.

## 2. Materials and Methods

### 2.1. Study Population

A total of 56 individuals carrying pathogenic or likely pathogenic sarcomeric variants were recruited from a dedicated familial HCM program. All participants provided written informed consent. The study was conducted in accordance with the Declaration of Helsinki [16], and approved by a single regional ethics committee, the Cantabria Research Ethics Committee (CEIC-IDIVAL; Santander, Spain. Ref. 2018.286).

### 2.2. Clinical Phenotyping

Participants underwent comprehensive clinical evaluation including medical history, electrocardiography, transthoracic echocardiography and cardiac magnetic resonance imaging when available. Diagnostic classification of HCM followed European Society of Cardiology criteria [2]. Interventricular septal thickness was selected a priori as the primary structural marker of disease expression.

### 2.3. Genetic Analysis

Genomic DNA was extracted from dried blood spot samples obtained by finger prick using the QIAamp^®^ DNA Mini Kit (Qiagen, Hilden, Germany), following the manufacturer’s instructions. Genotyping of the *SLC16A1* (MCT1) rs1049434 polymorphism (T1470A; Asp490Glu) was performed by real-time polymerase chain reaction using a TaqMan^®^ SNP Genotyping Assay (Applied Biosystems, Foster City, CA, USA) on a StepOne™ Real-Time PCR System (Applied Biosystems). Appropriate positive and negative controls were included on each plate to ensure genotyping accuracy. Genotype and allele frequencies were tested for Hardy–Weinberg equilibrium.

### 2.4. Exercise Classification

Habitual vigorous exercise was defined as regular participation in competitive or high-intensity endurance sports, corresponding to sustained activity above 6 metabolic equivalents for several hours per week over multiple years, consistent with competitive-athlete frameworks and eligibility recommendations [17,18].

### 2.5. Statistical Analysis

Continuous variables are presented as mean ± standard deviation and categorical variables as counts and percentages. Comparisons between groups were performed using Student’s *t*-test or non-parametric equivalents for continuous variables, and chi-square or Fisher’s exact tests for categorical variables. Statistical significance was set at *p* < 0.05. Given the exploratory nature of the study, analyses were interpreted cautiously without multivariable adjustment.

## 3. Results

### 3.1. Cohort Characteristics

Among the 56 sarcomeric variant carriers, 26 individuals (10 women and 16 men) fulfilled diagnostic criteria for HCM. Pathogenic variants were distributed across the principal genes implicated in HCM, including *MYBPC3*, *MYH7*, *ACTC1*, *MYL3*, *TNNI3*, and *TTN*. Baseline characteristics of the full cohort are summarized in Table 1. Genotype distributions conformed to Hardy–Weinberg equilibrium in the overall cohort and in sex-stratified analyses for both the full carrier cohort and the hypertrophic cardiomyopathy subgroup. A complete list of variants is provided in the Supplementary Tables.

### 3.2. Sex-Specific Metabolic–Phenotypic Interaction

In women with HCM, carriers of the MCT1 rs1049434 T-allele (TT or TA) exhibited significantly greater interventricular septal thickness compared with AA homozygotes (23.2 ± 6.8 vs. 14.2 ± 2.6 mm; *p* = 0.037) (Table 2). This difference was observed in the absence of significant disparities in age or age at diagnosis, suggesting a modifying role of metabolic genotype on structural remodeling. In contrast, no genotype-dependent differences in septal thickness were observed among men.

### 3.3. Influence of Vigorous Physical Activity on Male HCM Severity

Among male patients with HCM, those engaged in habitual vigorous exercise showed a directionally milder phenotype, characterized by lower septal thickness and a more favorable hemodynamic and tissue profile, including a lower prevalence of left ventricular outflow tract obstruction, less frequent left atrial enlargement, and a reduced burden of myocardial fibrosis on imaging (Table 3). Although these differences did not reach statistical significance (septal thickness: 18.3 ± 4.1 vs. 19.9 ± 6.9 mm; *p* = 0.585), the overall pattern consistently favored trained individuals.

### 3.4. Arrhythmic Burden and Event-Free Survival

Arrhythmic events were infrequent in this cohort. Neither MCT1 genotype nor vigorous exercise was associated with an increased arrhythmic burden. Given the limited number of events, these observations should be interpreted as descriptive.

## 4. Discussion

This study provides evidence that the phenotypic impact of the MCT1 rs1049434 polymorphism in sarcomere-positive HCM is sex-dependent and context-specific. In women, the presence of the T allele of the MCT1 rs1049434 polymorphism—previously associated with reduced lactate transport efficiency—is related to greater septal hypertrophy. This observation is consistent with experimental models and human exercise studies showing increased glycolytic strain in carriers of this variant [9,11]. These findings integrate well with recent multi-omics analyses of human HCM myocardium showing profound metabolic remodeling characterized by enhanced glycolysis, reduced fatty acid oxidation and altered redox balance [5].

In men, no direct effect of MCT1 genotype on structural expression was observed. Notably, habitual vigorous exercise was associated with a more favorable phenotype, suggesting that metabolic conditioning enhances oxidative capacity and lactate utilization, thereby mitigating the energetic consequences of sarcomeric dysfunction [11,12,13,14]. These findings were interpreted in the context of comprehensive multimodality cardiac imaging, which is essential to distinguish physiological athletic remodeling from pathological hypertrophy in HCM, as emphasized in recent imaging-based reviews [19]. While these observations are exploratory, they align with emerging data supporting the safety of carefully monitored physical activity in selected patients with HCM [14,15].

Importantly, genotype distribution analyses indicate that MCT1 rs1049434 does not influence disease penetrance but rather modulates phenotypic severity once HCM is established. This distinction reinforces the concept of MCT1 as a modifier gene rather than a primary determinant of disease.

A plausible mechanistic framework linking rs1049434 to sex- and exercise-dependent remodeling in HCM involves altered lactate flux within the cardiac microenvironment. Recent evidence supports a cell-to-cell lactate shuttle in the heart, in which cardiomyocytes predominantly express MCT1, whereas cardiac fibroblasts preferentially express MCT4, potentially supplying lactate to neighboring cardiomyocytes under stress conditions [20]. Perturbations that increase local lactate availability have been shown to promote hypertrophic responses and modulate cardiomyocyte gene expression programs [20,21]. In this context, rs1049434—previously associated with altered MCT1-mediated lactate transport—could contribute to an energetic mismatch in genetically predisposed individuals, thereby amplifying hypertrophic signaling.

Sex-related differences in myocardial substrate utilization may further modulate this effect. Prior human imaging studies have reported sex differences in myocardial oxidative metabolism and glucose utilization, suggesting that female myocardium may rely on distinct metabolic pathways under comparable workloads [22]. Therefore, it is biologically plausible that impaired monocarboxylate handling could have a greater phenotypic impact in women, consistent with the greater septal hypertrophy observed in female T-allele carriers in our cohort. Supporting the relevance of lactate signaling to hypertrophic remodeling, Wei et al. reported increased lactate uptake in hypertrophied cardiomyocytes, accompanied by upregulation of MCT1 and hypertrophy-associated genes, and showed that disruption of this lactate axis attenuated hypertrophy-linked transcriptional responses [23].

Conversely, exercise-induced metabolic conditioning may partially buffer lactate-related energetic stress in men. Vigorous training enhances oxidative capacity and metabolic flexibility, which may mitigate the structural expression of sarcomeric energetic inefficiency, aligning with the directionally milder phenotype observed in trained men in our cohort. Importantly, these mechanistic considerations remain hypothesis-generating in the absence of direct myocardial metabolic measurements in the present study and should be tested in larger cohorts and studies incorporating cardiac metabolic profiling.

Collectively, these findings support a three-way interaction between metabolic genotype, biological sex and environmental conditioning. In women, reduced efficiency of monocarboxylate handling associated with the MCT1 T-allele may exacerbate energetic stress and hypertrophic signaling, consistent with lactate/pyruvate-axis and fibroblast–cardiomyocyte lactate-shuttle mechanisms described in experimental models [23,24]. In men, greater metabolic reserve and exercise-induced mitochondrial adaptation appear to buffer these effects, resulting in attenuated structural remodeling.

Before drawing final conclusions, several methodological aspects should be acknowledged when interpreting these results. First, the exploratory nature of this study, together with the limited sample size, warrants cautious interpretation of the findings, which should be confirmed in larger, independent cohorts. In addition, no direct measurements of circulating lactate levels, systemic acid–base status, or myocardial metabolic flux were available. Consequently, mechanistic interpretations regarding lactate handling are inferred from established functional data on the MCT1 rs1049434 polymorphism and should be considered hypothesis-generating. Finally, the absence of direct myocardial metabolic or functional validation further constrains mechanistic inference and should be addressed in future investigations.

Within this interpretative framework, the MCT1 rs1049434 variant does not seem to act as a universal determinant of disease severity, but rather as a context-dependent modifier, with phenotypic impact emerging under specific biological conditions—most notably in female myocardium and in the absence of exercise-enhanced oxidative capacity. In women, the T-allele is associated with more pronounced septal hypertrophy, consistent with heightened susceptibility to energetic stress. In men, particularly those engaged in vigorous physical activity, exercise-induced metabolic conditioning appears to attenuate disease expression. This genotype–sex–environment interplay offers a plausible mechanistic framework for the observed heterogeneity of HCM remodeling and reinforces the relevance of precision-medicine approaches integrating metabolic genetics with individualized lifestyle considerations to refine risk stratification and clinical management.

## Figures and Tables

**Table 1:** Baseline characteristics of the full cohort of sarcomere variant carriers.

Variable	Total Cohort (*n* = 56)
Age, years	32 ± 17 (range 5–65)
Female sex	27 (48.2%)
Male sex	29 (51.8%)
Sarcomeric variant carriers with HCM	26 (46.4%)
Sarcomeric variant carriers without HCM	30 (53.6%)
Vigorous physical activity	14 (25.0%)
Non-athletes	42 (75.0%)
Index cases	12 (21.4%)
Relatives identified by cascade screening	44 (78.6%)
Main sarcomeric genes involved	*MYBPC3, MYH7, ACTC1, MYL3, TNNI3, TTN*

Data are expressed as mean ± standard deviation (range) or number (%). Vigorous physical activity was defined as habitual engagement in competitive or high-intensity endurance sports. A complete list of sarcomeric variants is provided in Supplementary Table S3. HCM: hypertrophic cardiomyopathy.

**Table 2:** Clinical and imaging characteristics of female patients with hypertrophic cardiomyopathy according to MCT1 rs1049434 genotype.

Variable	All Women with HCM (*n* = 10)	TT/TA Genotype (*n* = 6)	AA Genotype (*n* = 4)	*p* Value
Current age, years	42 ± 10	46 ± 7	37 ± 14	0.209
Age at diagnosis, years	32 ± 13	32 ± 15	32 ± 11	1.00
Maximal interventricular septal thickness, mm	19.6 ± 7.0	23.2 ± 6.8	14.2 ± 2.6	0.037
Maximal posterior wall thickness, mm	9.8 ± 3.2	10.3 ± 4.6	9.3 ± 1.3	0.688
Hypertrophy pattern				0.999
–Apical	1 (10%)	1 (16.7%)	0 (0%)	
–Asymmetric septal	8 (80%)	4 (66.7%)	4 (100%)	
–Concentric	1 (10%)	1 (16.7%)	0 (0%)	
Abnormal ECG	5 (50%)	4 (66.7%)	1 (25%)	0.524
LV outflow tract obstruction	3 (30%)	3 (50%)	0 (0%)	0.200
Systolic dysfunction	2 (20%)	2 (33.3%)	0 (0%)	0.467
Diastolic dysfunction	8 (80%)	5 (83.3%)	3 (75%)	1.00
Left atrial dilation (≥45 mm)	4 (40%)	3 (50%)	1 (25%)	0.571
Myocardial fibrosis on CMR	2 (25%)	2 (50%)	0 (0%)	0.429
Any arrhythmia	4 (40%)	2 (33.3%)	2 (50%)	1.00

Data are expressed as mean ± SD or number (%). Septal thickness refers to maximal wall thickness. CMR: cardiac magnetic resonance. LV: left ventricular.

**Table 3:** Clinical and imaging characteristics of male patients with hypertrophic cardiomyopathy according to habitual vigorous physical activity.

Variable	All Men with HCM (*n* = 16)	Athletes (*n* = 12)	Non-Athletes (*n* = 4)	*p* Value
Current age, years	40 ± 16	40 ± 16	39 ± 15	0.914
Age at diagnosis, years	28 ± 11	27 ± 11	30 ± 13	0.657
Maximal interventricular septal thickness, mm	18.7 ± 4.8	18.3 ± 4.1	19.9 ± 6.9	0.585
Maximal posterior wall thickness, mm	12.4 ± 2.9	12.2 ± 2.2	13.0 ± 4.7	0.652
Hypertrophy pattern—asymmetric septal	10 (66.7%)	8 (72.7%)	2 (50%)	0.564
Abnormal ECG	16 (100%)	12 (100%)	4 (100%)	1.00
LV outflow tract obstruction	3 (18.7%)	1 (8.3%)	2 (50%)	0.136
Systolic dysfunction	4 (25%)	3 (25%)	1 (25%)	1.00
Diastolic dysfunction	11 (68.7%)	8 (66.7%)	3 (75%)	1.00
Left atrial dilation (≥45 mm)	6 (37.5%)	4 (33.3%)	2 (50%)	0.604
Myocardial fibrosis on CMR	8 (72.7%)	5 (71.4%)	3 (75%)	1.00
Any arrhythmia	5 (31.2%)	3 (25%)	2 (50%)	0.547

Data are expressed as mean ± SD or number (%). Athletes were defined as individuals engaged in habitual vigorous physical activity. CMR: cardiac magnetic resonance. ECG: electrocardiogram. LV: left ventricular.

## Data Availability

The data supporting the findings of this study are publicly available in the University of Cantabria Institutional Repository at https://repositorio.unican.es/xmlui/handle/10902/34700 (accessed on 25 December 2025).

## Supplementary Materials

The following supporting information can be downloaded at: https://www.mdpi.com/article/10.3390/genes17020188/s1. Table S1. Genotype distribution and allele frequencies of the MCT1 rs1049434 polymorphism in the full cohort of sarcomeric variant carriers; Table S2. Genotype distribution and allele frequencies of the MCT1 rs1049434 polymorphism in patients with hypertrophic cardiomyopathy; Table S3. Sarcomeric gene variants identified in the cohort of sarcomeric variant carriers; Table S4. Sarcomeric gene variants identified in patients with hypertrophic cardiomyopathy. Refs. [25,26,27,28,29,30,31,32,33,34,35,36,37,38,39,40,41,42,43] are cited in Supplementary Materials.

## Abbreviations

The following abbreviations are used in this manuscript:

CMR	cardiac magnetic resonance
ECG	electrocardiogram
HCM	hypertrophic cardiomyopathy.
LVOT	left ventricular outflow tract
MCT1	monocarboxylate transporter 1
