# Supplementary material for: Sex- and Exercise-Dependent Modulation of Hypertrophic Remodeling by the MCT1 rs1049434 Polymorphism
Source: Genes (Basel). 2026 Feb 2;17(2):188. doi: 10.3390/genes17020188 (PMC12940516; doi:10.3390/genes17020188)
Supplement: Supplementary file 1 [file genes-17-00188-s001.zip › genes-4094255-supplementary.pdf]

Supplementary data

Table S1. Genotype distribution and allele frequencies of the MCT1 rs1049434 polymorphism in the full cohort of sarcomeric variant carriers.

Genotype / Allele	Total (*n* = 56)	Women	Men
TT	9 (16.1%)	7 (25.9%)	2 (6.9%)
TA	27 (48.2%)	14 (51.9%)	13 (44.8%)
AA	20 (35.7%)	6 (22.2%)	14 (48.3%)
**T allele**	45 (0.40)	28 (0.50)	17 (0.30)
**A allele**	67 (0.60)	26 (0.50)	41 (0.70)

Data are presented as *n* (%) for genotypes and *n* (allele frequency) for alleles. Genotype distributions conformed to Hardy–Weinberg equilibrium.

Table S2. Genotype distribution and allele frequencies of the MCT1 rs1049434 polymorphism in patients with hypertrophic cardiomyopathy (n = 26)

Genotype / Allele	Total (*n* = 26)	Women (*n* = 10)	Men (*n* = 16)
TT	3 (11.5%)	1 (10.0%)	2 (12.5%)
TA	13 (50.0%)	5 (50.0%)	8 (50.0%)
AA	10 (38.5%)	4 (40.0%)	6 (37.5%)
**T allele**	19 (0.37)	7 (0.35)	12 (0.37)
**A allele**	33 (0.63)	13 (0.65)	20 (0.63)

Data are presented as *n* (%) for genotypes and *n* (allele frequency) for alleles. Genotype distributions conformed to Hardy–Weinberg equilibrium.

Table S3. Sarcomeric gene variants identified in the cohort of sarcomeric variant carriers (*n* = 56)

Gene	Variant (HGVS)	rs number	Clinical significance	Reference	No. of patients
ACTC1	NP_005150.1:p.Leu10Met	rs397517057	Likely pathogenic	Kindel et al. 2012, [25]	2
MYBPC3	NM_000256.3:c.2308+1G>A	rs112738974	Pathogenic	Carrier et al. 1997, [26]	2
MYBPC3	NM_000256.3:c.505+5G>C	rs727503219	Likely pathogenic	Lin et al. 2010, [27]	1
MYBPC3	NP_000247.2:p.Arg1241fs		Pathogenic		1
MYBPC3	NP_000247.2:p.Arg820Trp	rs775404728	Pathogenic	Ripoll Vera et al. 2010, [28]	2
MYBPC3	NM_000256.3:c.906-36G>A	rs864622197	Pathogenic	Erdmann et al. 2003, [29]	2
MYBPC3	NP_000247.2:p.Ser406Asn	rs1053965064	Likely pathogenic	Gómez et al. 2017, [30]	1
MYBPC3	NP_000247.2:p.Val931Glyfs*120	----	Pathogenic	Gómez et al. 2014, [31]	18
MYBPC3	NP_000247.2:p.Glu542Gln	rs121909374	Pathogenic	Carrier et al. 1997, [26]	2
MYBPC3 + *MYBPC3*	NP_000247.2:p.Gln969Ter<br>NP_000247.2:p.Gln998Glu	rs397515992<br>rs11570112	Pathogenic<br>Benign	Yu et al. 1998, [32]	4

MYBPC3 + *TNNT2*	NP_000247.2:p.Val931Glyfs*120<br>NP_001001430.1:p.Arg286His	----<br>rs141121678	Pathogenic<br>VUS	Van Driest et al. 2004, [33] Gómez et al. 2014, [31]<br>Ripoll-Vera 2016, [34]	2
MYBPC3 + *MYH6*	NP_000247.2:p.Val931Glyfs*120<br>NP_002462.2:p.Leu120Phe	----<br>rs769074801	Pathogenic<br>VUS	Gómez et al. 2014, [31]	2
MYBPC3 + *MYL2*	NP_000247.2:p.Val931Glyfs*120<br>NM_000432.4:c.354-3C>G	----<br>rs758158083	Pathogenic<br>VUS	Gómez et al. 2014, [31]<br>Restrepo-Córdoba et al. 2017, [35]	1
MYBPC3 + *RBM20*	NP_000247.2:p.Val931Glyfs*120<br>NP_001127835.2:p.Asp888Asn	----<br>rs201370621	Pathogenic<br>Benign	Gómez et al. 2014, [31]<br>Refaat et al. 2012, [36]	1
MYH7	NP_000248.2:p.Asn479Ser	rs727504236	Pathogenic	Richard et al. 2003, [37]	4
MYH7	NP_000248.2:p.Asp1272Asn	rs730880906	Likely pathogenic		1
MYH7	NP_000248.2:p.Ile263Thr	rs397516269	Pathogenic	Tesson et al. 1998, [38]	1
MYH7	NP_000248.2:p.Ala797Thr	rs3218716	Pathogenic	Moolman et al. 1995, [39]	1
MYL3	NP_000249.1:p.Glu143Lys	rs104893750	Likely pathogenic	Olson et al. 2002, [40]	3
MYL3	NP_000249.1:p.Met173Val	rs199474708	Likely pathogenic	Morita et al. 2008, [41]	1
TNNI3	NP_000354.4:p.Arg162Trp	rs368861241	Pathogenic	Kimura et al. 1997, [42]	2
TNNI3	NP_000354.4:p.Ser199Asn	rs730881091	Pathogenic	Mogensen et al. 2004, [43]	1
TTN	NP_0033101.4:p.Glu11686*	Likely pathogenic	Likely pathogenic		1

Table S4. Sarcomeric gene variants identified in patients with hypertrophic cardiomyopathy (*n* = 26)

Gene	Variant (HGVS)	rs number	Clinical significance	Reference	No. of patients
MYBPC3	NM_000256.3:c.505+5G>C	rs727503219	Likely pathogenic	Lin et al., 2010, [27]	1
MYBPC3	NP_000247.2:p.Arg1241fs	—	Pathogenic	—	1
MYBPC3	NM_000256.3:c.906-36G>A	rs864622197	Pathogenic	Erdmann et al., 2003, [29]	2
MYBPC3	NP_000247.2:p.Ser406Asn	rs1053965064	Likely pathogenic	Gómez et al. 2017, [30]	1
MYBPC3	NP_000247.2:p.Val931Glyfs*120	—	Pathogenic	—	8

MYBPC3	NP_000247.2:p.Glu542Gln	rs121909374	Pathogenic	Carrier et al. 1997, [26]	1
MYBPC3<br>+<br>*MYBPC3*	NP_000247.2:p.Gln969Ter<br>NP_000247.2:p.Gln998Glu	rs397515992<br>rs11570112	Pathogenic<br>Benign	Yu et al. 1998, [32]<br>Van Driest et al. 2004, [33]	1
MYBPC3<br>+<br>*TNNT2*	NP_000247.2:p.Val931Glyfs*120<br>NP_001001430.1:p.Arg286His	----<br>rs141121678	Pathogenic<br>VUS	Gómez et al. 2014, [31]<br>Ripoll-Vera 2016, [34]	1
MYBPC3<br>+<br>*MYL2*	NP_000247.2:p.Val931Glyfs*120<br>NM_000432.4:c.354-3C>G	----<br>rs758158083	Pathogenic<br>VUS	Gómez et al. 2014, [31]<br>Restrepo-Córdoba et al. 2017, [35]	1
MYH7	NP_000248.2:p.Asn479Ser	rs727504236	Pathogenic	Richard et al. 2003, [37]	2
MYH7	NP_000248.2:p.Asp1272Asn	rs730880906	Likely pathogenic		1
MYH7	NP_000248.2:p.Ile263Thr	rs397516269	Pathogenic	Tesson et al. 1998, [38]	1
MYH7	NP_000248.2:p.Ala797Thr	rs3218716	Pathogenic	Moolman et al. 1995, [39]	1
MYL3	NP_000249.1:p.Glu143Lys	rs104893750	Likely pathogenic	Olson et al. 2002, [40]	1
MYL3	NP_000249.1:p.Met173Val	rs199474708	Likely pathogenic	Morita et al. 2008, [41]	1
TNNI3	NP_000354.4:p.Ser199Asn	rs730881091	Pathogenic	Mogensen et al. 2004, [43]	1
TTN	NP_0033101.4:p.Glu11686*	Likely pathogenic	Likely pathogenic		1
